# Developing Measures of Watershed Knowledge

**DOI:** 10.1007/s00267-026-02436-x

**Published:** 2026-04-09

**Authors:** Stephen Mainzer, James Price Dillard

**Affiliations:** 1 Landscape Architecture, The Pennsylvania State University, University Park, PA USA; 2 Communication Arts & Sciences, The Pennsylvania State University, University Park, PA USA

**Keywords:** Chesapeake Bay, Pennsylvania, Watershed, Collective identity, Water quality, Index development

## Abstract

The Chesapeake Bay Watershed has faced long-standing challenges to water quality, a condition that impacts its residents access to clean air, water, and economic status. We suggest that collective action is necessary by watershed residents to address this problem. But, before collective action may be encouraged, we must first reliably measure the four stages of collective identity. Here, we briefly review the theoretical foundations for constructing tools that measure the first two stages: knowledge of the group and knowledge of group membership, or in the case of a watershed, general and local knowledge. Results from a survey of Pennsylvania residents show that measuring such knowledge is possible and that there are key differences between general and local knowledge. We discuss practical applications for watershed managers and describe options for shortening the indices.

## Introduction

Large-scale natural resources that cross civic boundaries face unique challenges to their long-term management. The longstanding ability for federal-agencies in the United States to interpret and enforce pro-environmental policies has been recently challenged. (Xiang 2024, Mainzer 2025). As federal capacity decreases, collective action is increasingly necessary by the affected residents that rely on such resources for access to clean water, clean air, and productive economics. The Chesapeake Bay Watershed exemplifies such a challenge. The watershed is a natural resource of substantial value shared by millions of people with unique social identities. Since 1983 it has experienced poor to moderate water quality (Mainzer et al. 2024, Chesapeake Bay Program 2024), placing a $22.5–$129.7 billion dollar agriculture, aquaculture, and recreation/tourism system at risk (Mueller 2024, p. 4). The watershed is a regional-scale natural resources whose geographic boundaries intersect with multiple civic boundaries. Such shared water resources are complex systems managed by transboundary governance at local and multi-state scales (Johns 2017 and Johns and VanNijnatten 2021), such as the Chesapeake Bay Foundation (Chesapeake Bay Watershed Agreement 2022), the Chesapeake Bay Program (Chesapeake Bay Program 2024), the federal Environmental Protection Agency (Mueller, 2024), and a Presidential Executive Order (Executive Order #13508 2009). Within the common need for clean water lies the potential for collective identity. In Mainzer et al. 2024, we created a stage-model to describe the development of the identity necessary for collective action, as seen through a case study of the Chesapeake Bay Watershed. Here, we devise some of the tools necessary to assess the stage components of that model. Developing precise and accurate measurement is a necessary step toward describing the current state of collective identity in the watershed. With such tools in hand, the success or failure of messaging efforts to create collective identity can be evaluated against baseline estimates of identity.

### A Stage Model of Collective Watershed Identity

Social identity theory and its offspring, social categorization theory, are premised on the notion that individuals explain and understand themselves in terms of their identities (Hornsey, 2008). This key concept—identity—answers the question: *Who am I?* Individuals may possess many identities (e.g., atheist, actor, ambulance driver) that vary in salience at any given moment. But, regardless of their number, however, all identities serve two functions: they assist individuals with the task of making sense of their environment and they provide guidance on how to behave (Weick, 2005). *Social* and *collective* identities are terms that reflect membership in a social group (cf., David and Bar-Tal 2009) (e.g., creed, country, color, class, or culture^1^). Social/collective identities exist to the extent that individuals jointly understand their boundaries and see themselves as sharing a common history and fate with other members of the group (Ashmore et al. 2004; David and Bar-Tal 2009). When these factors are in place, individuals experience a sense of groupness and may behave in ways that promote the well-being of fellow category members.

Central to the current undertaking, collective identities can be placed-based. For instance, individuals who reside within the boundaries of the state of Pennsylvania are members of the group known as Pennsylvanians. Strictly speaking, this is also true of the Chesapeake Bay Watershed, although we suspect that individuals may be less confident of their membership in the watershed or even completely unaware of it. A watershed that supports 23 million people (Manson et al. 2022) is, necessarily, a socially heterogenous group. Individuals who reside in large groups will hold multiple identities that may or not align with each other. For example, country-level partisan groups may share group-based social identities of social class, race, ethnicity, and religious background (Hopkins 2018, p. 8). But partisan groups need not be homogenous. Likewise, despite differences in individual-level identities, access to clean water affects all watershed residents, albeit in different ways. It is therefore equally possible that some shared identities may cut across heterogenous groupings. Whether a watershed-based identity can be shared across a heterogenous group as large as a U.S. state is a question of whether individuals hold the prerequisite knowledge and share a sense of interdependence with the group.

This does not change the fact that the problems of the Chesapeake Bay are problems of collective action. Mainzer et. al. (2025) suggests that addressing those ecological challenges requires widespread development of a collective watershed identity. Our stage model of identity development was designed to provide theoretical guidance to applied efforts to create such an identity. It proposes that individuals can be grouped according to their membership in one of five distinct and ordered categories. Movement from one stage to the next involves stage-specific changes that can, in principle, be achieved via persuasive messaging (Weinstein et al. 1998). Next, we describe each stage in general, theoretical terms as well as watershed-specific terms.

*Stage 0, Unawareness*: In this phase, individuals have little or no knowledge that the group exists because they are unfamiliar with the defining features of the group. With respect to watersheds, some segments of the population may have little or no understanding of what watersheds are, how they function, or how they impact human existence.

*Stage 1, Knowledge of the Group*: Individuals who fall into this stage are aware of a group’s existence and the principle by which its boundaries demarcate individuals as members or nonmembers of the group. Because watersheds are placed-based phenomena, individuals need to understand basic principles of physics (e.g., water flows downhill) and geography (e.g., land exists at different elevations) as they pertain to watersheds. The boundary principle is abstract knowledge that might (in the next stage) be called upon to address the question of whether or not any given individual is a group member. We refer to this as *general* knowledge.

*Stage 2, Knowledge of Group Membership:* Fominaya (2010) writes that “the process of establishing what ‘we are’ inevitably involves establishing what ‘we are not” (p. 395). Individuals in Stage 2 have moved beyond abstract knowledge of group membership to specific knowledge as their own in-group or out-group status as residents of the watershed. This observation is complicated by the nesting-doll aspect of watersheds. Although our primary focus is on the Chesapeake Bay Watershed, the physical boundaries of any given watershed subsume smaller watersheds and are subsumed by superordinate watersheds (up to a point). The question of whether an individual resides inside or outside of a particular watershed locates the person with respect to the group. We refer to this as *local* knowledge.

*Stage 3, Engagement*: Individuals may believe that they share a common set of values with other group members, though not necessarily common experiences. An analogy here is the individual who endorses pro-environmental positions, but has not (yet) directly experienced climate-induced flooding, hurricanes, extreme temperature change, or any of the other sequelae of climate change. Thus, engagement occurs in the abstract.

*Stage 4, Embrace:* A stronger form of collective identity follows from shared experiences, either past or anticipated. When individuals simultaneously undergo events that are consequential to their well-being, they become motivated to develop a shared interpretation of those events. Both the process and product of sensemaking contribute to a heightened feeling of collectivity. In this vein, it is often held that natural disasters can increase levels of social cohesion among members of affected communities (e.g., Calo-Blanco et al. 2017). Similarly, direct, shared experience with the problems of watershed mismanagement should amplify embrace of a watershed identity. We suggest that even the anticipation of shared consequence is sufficient to produce Embrace.

### Evaluating the Stage Model of Collective Watershed Identity

Stage models are such that (a) is each stage constitutes a qualitatively distinct category and (b) the categories are ordered—each stage is a necessary developmental condition for the stage that follows (Weinstein et al. 1998). Thus, different stages possess different properties. In the current context, it is clear that stages 0-2 can be distinguished from one another in terms of differences in knowledge. The model holds that people in Stage 0 lack knowledge, those in Stage 1 possess general knowledge, and those in Stage 2 hold general *and* local knowledge about their group membership (i.e., their location with respect to watershed boundaries). Stages 3 and 4 are also clearly different from one another, though the basis for distinguishing between them hinges on differences in the way that they interface with watershed issues. Stage 3 is premised on abstract values, whereas Stage 4 is the product of abstract values in combination with concrete experience, past or anticipated.

Given the differences among the stages, devising measures capable of discriminating among them is a complex task requiring multiple studies. In this project, we focused attention on the *general* and *local* knowledge, which allows discrimination of stages 0, 1, and 2. Our overarching goal was to design and validate indices for assessing general and specific indices of watershed knowledge. Such measures are necessary for empirical efforts to assess the validity of the theoretical model. They can also provide insight into the levels of watershed knowledge present in any given population. This information would clarify the challenges faced by watershed managers seeking to educate citizens by allowing managers to meet them where they are.

#### The Meaning of Knowledge

Although *knowledge* has a seemingly simple meaning, parsing it may prove instructive. First, when individuals can accurately identify or articulate agreed-upon facts, we say that they are right or correct. As students and teachers know, there are degrees of rightness and that variation is reflected in exam scores. Some people know more than others (e.g., Stage 0 vs. Stage 1). Being wrong is different because there is more than one way to be incorrect. Perhaps an individual cannot recall the information needed to respond to an essay question. The answer is marked wrong because it is blank. A different type of error occurs when individuals cannot discriminate statements that are true from those that are false. It is not that knowledge is absent, rather incorrect knowledge is present. This difference is roughly analogous information versus misinformation. Because neither type of error is desirable to promoting accurate understanding of watersheds and they might require different sorts of interventions to increase accuracy. Thus, we aimed to assess the degree to which each was present. More formally, we asked:

*RQ1: To what extent do the data show evidence of (a) inaccurate beliefs and/or (b) lack of accurate beliefs about watersheds?*

#### Establishing the Construct Validity of a Watershed Knowledge Index

Scientific theories require valid measures if empirical tests of their predictions are to be meaningful. It is often said that indices are valid when they measure what they are supposed to measure. Fortunately, there are concrete, well-established procedures for assessing the degree to which an index achieves the goal of measuring what it is supposed to measure. Cronbach and Meehl’s (1955) arguments for the utility of nomological networks—a web of empirical relationships between the focal construct and other theoretically-relevant constructs—were foundational to assessing construct validity. A more sophisticated version of earlier ideas was articulated in Hunter and Gerbing (1982). These latter authors propose three necessary criteria, which, when taken together are sufficient to make a compelling argument for validity.

*Substantive validity* refers to a judgment regarding the meaning of the empirical indicators of the focal construct(s). Such judgments have, at minimum, two aspects: Content and scope. Assessing items for appropriate content involves a conceptual evaluation of the degree to which the item’s meaning corresponds with that of the latent construct. Assessments of scope are attentive to the conceptual boundaries of the focal concept. It should not go unnoted that judgments are made by people. One might turn to subject matter experts (SMEs) to render decisions as to content and scope because they possess sophisticated knowledge of the conceptual domain. Of equal importance, however, are the respondents themselves. The opinions of SMEs are unimportant if the items are not understandable to the population of interest.

As used by Hunter and Gerbing (1982), *internal consistency* means that items intended to measure the same construct behave similarly to one another (cf., Campbell and Fiske’s (1959) convergent validity). More concretely, it refers to the comparability in sign and magnitude of the item loadings on their latent factor. Hence, internal consistency is an item-level judgment. This usage is distinct from coefficients of reliability, such as Cronbach’s alpha, which summarize associations among indicators.

*Parallelism* is the notion that items that truly measure the same construct should show parallel relationships with other constructs. In Hunter and Gerbing’s (1982) exposition of confirmatory factor analysis items are parallel when they exhibit a pattern of factor loadings on theoretically-relevant constructs that are similar in both sign and magnitude. As with Campbell and Fiske’s (1959) nomological network, the strength of this test depends on the size and quality of the set of reference variables.

We designed two studies to evaluate the construct validity of our watershed knowledge measures and asked:

*RQ2: To what degree do the data show evidence of substantive validity, internal consistency, and parallelism for the general and specific measures of watershed knowledge?*

The frequent need to measure social scientific constructs at scale pits two dynamics against one another. Because reliability sets a ceiling on validity, researchers should use the most comprehensive version of the measure whenever possible. However, large surveys consume the resources of both respondents and researchers, who provide time and money, respectively. These constraints often mean that only a limited number of items can be included. Consequently, it is common to see work devoted to the development of short-form indices of the constructs of interest. Rather than produce a single short form, we aimed to provide quantitative estimates of the tradeoff between the length and reliability of our knowledge instruments. This information will allow future researchers to make decisions that optimize the cost-validity tradeoff in the context of their own investigations.

*RQ3: What is the relationship between index length and reliability in the measures of watershed knowledge?*

We investigated these research questions via three studies. For Study 1, we sought to develop a bank of items for the measurement of *general* and *local* watershed for use with the general public. Our efforts were adapted to the Chesapeake Bay Watershed. Studies 2 and 3 were based on a survey administered to 400 Pennsylvania residents. Approximately half of Pennsylvania is located within the Chesapeake Bay Watershed. For Study 2, we evaluated the construct validity of the general and specific knowledge measures. In Study 3, we examined the trade-offs of a short-form index and reliability.

## Method

### Study 1: Initial Design of the Watershed Knowledge Index (WKI)

#### Instrument Design

The study team constructed a preliminary bank of items based on their knowledge of the topic. Four items on general scientific knowledge were designed to ease participants into the survey and familiarize them with the structure of subsequent questions. The items concerned with general watershed knowledge covered three types of content: (a) two that defined watershed, (b) four items focused on the basic spatial concepts that define watersheds, and (c) seven items addressed the functions of watersheds. The warm-up and definition items offered four response options: One correct, two incorrect, and *I don’t know*. These options allowed us to measure the proportion of respondents who held correct beliefs versus incorrect beliefs versus uncertain knowledge. To reduce variance attributable to common method, the function items were true-false with an *I don’t know* option.

The two local knowledge items used different formats to ascertain the degree to which respondents knew their location vis a vis different watersheds. The first of these was an open-ended, unaided-recall question that asked respondents to write out the watersheds in which they resided. The second was an aided-recall, multiple choice question with six substantive options. Given our plan to sample only Pennsylvania residents, two options were factually incorrect and four were possibly correct. This item also included a seventh option: *I don’t know* to assess uncertainty. Participants could check as many options as applied to them.

#### Subject Matter Experts: Participants and Procedures

A subject matter expert (SME) was defined loosely as any individual with knowledge of watersheds that likely exceeded that of the general public. Operationally, we relied on formal credentials (i.e., possession or pursuit of topic-relevant degree) or employment in a non-academic, but ecologically-oriented position. Through the study team’s personal network and the email listservs of allied fields, SMEs were recruited to evaluate an initial draft of the WKI. Nine SMEs agreed to review the WKI and six completed their review over an average of 14 days. A summary of the experts’ qualifications is given in Table 1.^2^

**Table 1:** Qualifications of subject matter experts (SMEs)

ID	Level of Education	Position	Expertise
SME1	Ph.D.	Director of Research, Biogeochemistry	Wetland ecology, Sea level rise
SME2	Ph.D.	Professor of Agricultural and Biological Engineering	Environmental hydrology
SME3	Ph.D.	Assistant Professor of Landscape Architecture	Global health
SME4	Masters	Wetland Restoration Specialist	Watershed Restoration and Planning
SME5	Masters (pursuing)	Graduate Student	Ecology
SME6	Ph.D.	Professor & Extension Specialist, of Wetland Ecology and Restoration	Tidal and inland wetland ecology

Each SME received a copy of the draft questionnaire, which included a preamble to survey participants (in the next study) as well as instruction to SMEs. They were asked to review the set of items with regard to *content* (the essential aspects of watersheds), *accuracy* (the factual correctness of questions and answers), and *expression* (clarity of word choice and phrasing). The full set of instructions for subject matter experts is given in Table 2.

**Table 2:** Instructions to subject matter experts (SMEs)

• We are trying to develop a measure of knowledge about watersheds that can be administered to the general public. Towards that end, we have created a set of questions, much as one would create a test for a class. This is the kind of test that might be used at the beginning of a class to establish a baseline, then again at the end to see if learning occurred.
• We are seeking your expert evaluation of each question on several dimensions: 1) CONTENT: Are the questions the right ones to ask about the concept of watersheds? Are we missing any essential aspects of what watershed means? 2) ACCURACY: Are the answers that we have marked as correct factually accurate? Are the answers indicated as incorrect factually inaccurate? (Bear in mind that the test is intended for the general public. People with advanced degrees might make finer distinctions than should be expected from the public.) 3) EXPRESSION: Are the questions and the response options phrased clearly? If you see ways that the language could be simplified or shortened, please describe how we could make appropriate changes.

### Study 2: Construct Validation of the Revised WKI

#### Participants and Procedures

Residents of Pennsylvania who were members of the Prolific research panel were invited to participate in exchange for compensation of $12 per hour. The Prolific research panel enabled screening criteria that ensured that the sample matched the state population in terms of gender and race/ethnicity. The web-based survey was distributed to a stratified sample of Prolific research participants. Data collection took place during March of 2025.

To ensure data quality, respondents were reviewed according to time, attention, and completeness. Respondents’ time to complete the survey was reviewed via the Prolific research panel interface. Then, respondents were dropped if they (a) showed evidence of straight-lining, (b) failed attention-check questions, or (c) contained excessive missing data. These procedures yielded a final sample of *N* = 374. Mean, median, and modal time to complete the survey in the final sample were 18 minutes, 34 seconds, 9 minutes, 12 seconds, and 6 minutes, 33 seconds, respectively. Table 3 summarizes the demographic makeup of the sample and compares it to state census data from 2023.

**Table 3:** Comparison of sociodemographic characteristics between state and sample

	Pennsylvania	Sample
Total Population	12,986,518	374
Race		
White	75.8%	80.5%
Black	10.7%	11.5%
Asian	3.7%	4.8%
Other	3.4%	3.2%
Ethnicity		
Hispanic^a^	8.4%	7.8%
Sex		
Male	49.3%	50.0%
Female	50.7%	50.0%
(Mean) Age^b^	41.1	42.2
Education^b^		
At least a HS diploma	91.9%	99.5%
At least a Bachelor’s degree	34.5%	54.5%

^a^Race and Ethnicity were measured with separate questions in our survey

^b^Characteristics not included in sampling quota

#### Measures

Stage 1 general knowledge items appear in Table 4 along with response options. Whereas Stage 1 items have answers rooted in abstract, factual knowledge, Stage 2 items (Table 5) assess specific geographical knowledge. The latter, thus, presented some logistical issues given that watersheds exist at different scales and are nested within each other. A person might be able to name a local, stream-based watershed, but not know the name of the larger regional watershed within which the stream is nested. Further, zip codes are civic boundaries and watersheds are physical boundaries—rarely do the two systems align. This made accuracy difficult for us to gauge, especially at smaller watershed scales. In response, the study team developed a database that linked all zip codes in Pennsylvania to each watershed from hydrologic unit X to zipcode X, based on the location of the center point of the zip code. This created a short list of possible correct responses for each zip code. Participants who were able to correctly identify at least one watershed (at any scale and with leniency toward spelling) were marked as correct.

**Table 4:** Watershed knowledge index items, stage 1: general knowledge

	Item	Response options
*General Definition*		
GD1	The word “watershed” refers to:	○ Area of land in which all the water flows downhill to a single location [CORRECT]. ○ A small building that houses a well. ○ How an animal removes water from its body. ○ I don’t know.
*Spatial Concepts*		
SC1	The boundaries of a watershed are determined by:	○ Politics. ○ The shape and slope of the land [CORRECT]. ○ Legal agreements among counties and states. ○ I don’t know.
SC2	Watersheds:	○ May be different sizes [CORRECT]. ○ Are almost always large enough to contain at least three lakes. ○ Are typically the same size. ○ I don’t know.
SC3*	The boundaries of a watershed:	○ Change on a daily basis. ○ Are permanently fixed. ○ Can change over thousands of years [CORRECT]. ○ I don’t know.
SC4	Watersheds are located:	○ Only on the top of mountains. ○ Near the ocean. ○ Everywhere [CORRECT]. ○ I don’t know.
*Watershed Function*		
WF1	Watersheds can provide a source of freshwater for human use.	○ True [CORRECT] ○ False ○ I don’t know
WF2	Recreational opportunities such as hiking, fishing, and boating can be found within watersheds.	○ True [CORRECT] ○ False ○ I don’t know
WF3*	Watersheds do not have cultural significance.	○ True ○ False [CORRECT] ○ I don’t know
WF4	The agriculture industry depends on the reliable sources of water that watersheds provide.	○ True [CORRECT] ○ False ○ I don’t know
WF5	Watersheds may contain habitats that filter pollutants that help to ensure cleaner water downstream.	○ True [CORRECT] ○ False ○ I don’t know
WF6	Because watersheds provide regular water flow, they harm biodiversity.	○ True ○ False [CORRECT] ○ I don’t know

*items subsequently deleted following the study team’s analysis in coordination with subject matter expert comments.

**Table 5:** Watershed knowledge index items, stage 2: local knowledge

	Item	Response options
LK2	Because bigger watersheds contain smaller watersheds, everyone lives within several watersheds. Can you name one or more of the watersheds in which you reside?	Open ended
LK3	Do you live in any of the following watersheds? Check all that apply:	○ Ohio watershed ○ Chesapeake Bay Watershed ○ Genesee watershed ○ Delaware watershed ○ Erie watershed ○ I don’t know

The survey also included a set of reference variables based on the most recent US Census sociodemographic data collection. There were items to assess gender, age (computed as date of birth subtracted from 2025), time residing in the current zip code (number of years), race, ethnicity, education, income, and political affiliation. Non-ordinal reference variables were recoded as follows: race, white = 1, non-white = 0, ethnicity, Hispanic = 1, and non-Hispanic = 0. Party affiliation was captured using a 6-point scale (Liberal Democrat = 1, Moderate Democrat = 2, Moderate = 3, Moderate Republican = 4, Conservative Republican = 5, No affiliation or Other Affiliation = 6). The six participants (1.6%) who chose the latter option were recoded as Moderate.

#### Statistical Power

Any evaluation of parallelism depends on accurately assessing the presence *and* absence of associations between the focal constructs and the reference variables. An estimate of statistical power is important to both. Following Funder and Ozer (2019), we considered three effect sizes (*r* = 0.10, 0.20. and 0.30) to represent small, medium, and large effects, respectively. Assuming two-tailed tests, alpha of 0.05, and *N* = 374, statistical power was 0.62, 0.98, and 0.99. Thus, there was a strong likelihood that the data would detect medium and large effects, but a good chance that small, genuine effects would not register as significant.

### Study 3: Shorter and Longer Forms of the Watershed Knowledge Index?

Because data gathering consumes resources, researchers often desire measures that are as short as possible. Given that the general knowledge measure consists of nine-items, it was reasonable to ask what costs in reliability (and, therefore, validity) might be incurred by abbreviating the item set. Rather than arbitrarily choose some number of items and designate it as the short form, we conducted a series of analyses aimed at providing survey designers with the information to make their own choices.

A series of regression analyses were conducted in which the dependent variable was the total of the nine indicators of general knowledge. Items were entered one at a time to produce short-form indices that varied in length and reliability. Given that the general definition item (GD) and the water boundary item (SC1) possessed the strongest substantive validity (see Results: Study 2), they were forced into every equation at step 1. For all subsequent models, the entry criterion was stepwise.

## Results

### Study 1: Initial Design of the Watershed Knowledge Index (WKI)

The experts provided substantial feedback: 45 comments and 10 recommended text changes. Comments were generally positive and focused on improving the clarity of word choices within each survey item. For example, the group indicated a clear preference for one of the two general definition items. In another case, the SMEs agreed that adding the word “slope” to a question was important to ensure that some degree of topographic knowledge was included. Expert feedback was also helpful in refining social and demographic questions.

On the basis of the experts’ comments, the study team modified some questions and discarded others. The product of those efforts appears in Tables 4 and 5. These items formed the core of the survey that was conducted in the next phase of the project. The team flagged two items as possibly needing further evaluation (spatial concept #2 and watershed function #3).

### Study 2: Construct Validation of the Revised WKI

#### RQ1: Accuracy and Uncertainty of Watershed Knowledge

The first research question focused on different types of knowledge errors. Attention to the *Correct* column in Table 6 reveals high levels of general knowledge regarding the definition of watersheds (88.2%), spatial concepts associated with watersheds (average 76.0% with a range from 63.3% to 86.1%), and watershed functions (average 79.3% with a range from 72.2% to 83.5%); all Stage 1 measures. Turning to the error columns (*Incorrect* and *I don’t know*) less than 10% of the sample endorsed incorrect beliefs regarding spatial concepts (item SC3 is the exception). Similarly, 6% or less chose factually inaccurate beliefs in the watershed function items. Considered against RQ1, these findings indicate that both types of errors were relatively infrequent, but lack of watershed knowledge is a more common problem than is the embrace of inaccurate beliefs.^3^

**Table 6:** Item-level responses, stage 1: general knowledge

Item Identifiers and Content Domain	I do not know	Incorrect	Correct	Std. Deviation Correct
*General Definition*				
GD1_C	8.1%	3.7%	88.2%	0.323
*Spatial Concepts*				
SC1_C	10.5%	3.4%	86.1%	0.347
SC2_C	15.2%	2.1%	82.7%	0.379
SC3_C*	20.7%	16.0%	63.3%	0.483
SC4_C	19.7%	7.9%	72.4%	0.447
*Watershed Functions*				
WF1_C	15.7%	1.6%	82.7%	0.379
WF2_C	17.3%	3.9%	78.7%	0.410
WF3_C*	21.3%	6.6%	72.2%	0.449
WF4_C	15.5%	2.4%	82.2%	0.383
WF5_C	15.0%	1.6%	83.5%	0.372
WF6_C	17.6%	6.0%	76.4%	0.425

*items deleted from subsequent analyses

Table 7 reports the accuracy and uncertainty results for local knowledge (Stage 2). Here we see much higher levels of error with only 45.0% of respondents able to provide accurate information regarding their location in any watershed. It should be noted, however, that this result is the product of unaided recall, which is the more difficult task of the two items. Still, fewer than half of the respondents (42.5%) were able to correctly identify their location when presented with the aided recall item. The next most common response was *I don’t know* at 39.4% followed by choice of an incorrect option at 18.1%. In relative terms, the answer to RQ1 for local knowledge mirrors the results for general knowledge: More Pennsylvania residents lack Stage 2 knowledge than believe factually incorrect Stage 2 information.

**Table 7:** Item-level responses, stage 2: local knowledge

Item identifiers and content domain	Mean	Std. Deviation
LK2_C		
I do not know	33.3%	
Incorrect	21.7%	
At least 1 correct	45.0%	0.498
1	39.8%	
2	4.1%	
3	1.1%	
LK3_C		
I do not know	39.4%	
Incorrect	18.1%	
Correct	42.5%	0.495

Notably, items SC3 (20.7%) and WF3 (21.3%) received higher percentages of *I don’t know* responses than other Stage 1 items. These two items were noted by SMEs as reflecting especially nuanced knowledge (SC3) or as seeming out of place in attempting to capture cultural dimensions of watersheds (WF3), whereas other items focus on hydrological and spatial dimensions. After triangulating across insights from SMEs, the survey respondents, and the study team, we resolved to exclude items SC3 and WF3 from further analysis. This decision purified the general knowledge index by increasing content homogeneity.

RQ1 asked broadly about construct validity. On the basis of SME data, general Pennsylvania population data, and our own evaluation of the items, we concluded that the Stage 1 and 2 item sets were substantively valid. This partially answered RQ1. For the subsequent analyses, we collapsed across inaccurate and I don’t know responses so as to score all of the items as either correct or not correct. Then we proceeded with statistical tests designed to assess internal consistency and parallelism.

#### RQ2: Internal Consistency and Parallelism

Our first effort at evaluating the dimensionality of the knowledge items relied on a principal axis factor analysis followed by oblimin rotation. This yielded two factors, which were readily interpreted as *general knowledge* and *local knowledge*. The loadings of Stage 1 items on the general factor and the loadings of Stage 2 items on the local factor were both generally uniform, and thus evidence of internal consistency. The low cross-loadings also offered some evidence of parallelism. However, stronger tests could be achieved using confirmatory factor analyses in conjunction with the reference variables, which constitute a nomological network.

Toward that end, we used the AMOS software to produce maximum likelihood estimates of the model parameters. Global fit of the two-factor model was assessed with multiple indices: Comparative Fit Index (CFI), standardized root mean residual (SRMR), root mean squared error of approximation (RMSEA), and the probability of close fit (PCLOSE). Preferred values of each index (Hu and Bentler 1998; 1999) are given in the left-most column of Table 8. One final index, the Akaike Information Criterion (AIC), was used to assess fit differences between models. Values in the 1–2 range indicate comparable fit, in 4–7 range possible comparability, and values greater than 10 constitute strong evidence that one model is superior to the other (the one with the smallest AIC) (Burnham and Anderson 2002). The χ^2^ statistic is reported, but not interpreted, given its known bias toward false positives (Brown 2015, p. 69). Local fit was assessed in terms of the magnitude of factor loadings, standardized residual covariances, and modification indices.

The initial two-factor model fit the data well on all indices except the CFI, which was slightly below the target value of 0.95. Inspection of the modification indices revealed that a statistically significant improvement could be achieved by allowing the error terms for spatial concepts items 2 and 4 to correlate. It should be noted that correlated errors can be viewed as model misspecification (Gerbing and Anderson 1984) and, therefore, undesirable. However, Hall, Snell, and Foust (1999) demonstrate that such concerns are misplaced when the indicators tap the same latent construct and they will be combined to create indices—as we planned to do. The resulting model produced excellent global fit on all indices (third column, Table 8). Accordingly, no further modifications were made. Factor loadings ranged from 0.55 to 0.70 for the general knowledge items and from 0.57 to 0.73 for the local knowledge items. Although these loadings could be viewed as modest, it is important to bear in mind that the items are dichotomous and, therefore, less reliable than continuous indicators would be. We judged them to be satisfactory. Standardized residual covariances were all less than 2.

**Table 8:** Global fit indices for the confirmatory factor models

Indices	Models		
	*Two factors*	*Two factors with correlated errors (items SC2 & SC4)*	*One factor with* *correlated errors* *(items SC2 & SC4)*
χ2 (df)	202.29 (115)	175.03 (114)	270.63 (124)
CFI	0.931	0.952	0.884
RMSEA (90% CI)	0.045 (0.035/0.055)	0.038 (0.026//049)	0.056 (0.047/0.065)
PCLOSE	0.777	0.968	0.124
SRMR	0.0381	0.036	0.046
AIC	390.294	365.038	440.632

*CFI* Comparative Fit Index, *RMSEA* root mean squared error of approximation, *90%CI* = 90% confidence interval, *PCLOSE* probability of close fit, *SRMR* standardized root mean residual, *AIC* Akaike Information Criterion.

For an explicit test of an alternative, we created a single-factor model such that all of the items—general and local—were treated as indicators of one latent construct. To ensure fair comparison, correlated errors were allowed for SC2 and SC4. As shown in the rightmost column of the table, this model failed with respect to the CFI but was acceptable on the remaining fit indices. However, direct comparison with the two-factor model clearly demonstrated the superiority of the latter: The χ^2^ difference 95.6 (*df* = 10), *p* < 0.001 indicated that the two-factor model showed significantly better fit. The AIC difference between models was 75.59, which far exceeds the value of 10 that constitutes strong evidence of a difference in favor of the two-factor model. In short, the two-factor model with correlated errors was clearly the best-fitting model of the options tested. But, with or without the correlated errors, the data returned two distinct factors that were consistent with expectations.

Having established the dimensionality of the knowledge items, we computed reliabilities using coefficient alpha. They were 0.84 for general knowledge and 0.58 for local knowledge. Although the first value is satisfactory, the second one is low. It reflects the fact that (a) local knowledge is assessed with only two items and (b) the correlation between them is only 0.42. Values of this magnitude are not unusual between items. For example, correlations among the Stage 1 items ranged from 0.23 to 0.51. Further, it is important to bear in mind that coefficient alpha is best understood as a lower bound estimate of a measure’s true reliability (Bollen 1989).

Further evidence of the viability of the two-factor solution can be found in Table 9, which displays the correlations between the two types of knowledge with the set of reference variables. A between-column comparison of the coefficients shows them to be notably different from one another. If the general and local knowledge factors were tapping the same underlying construct, the pattern of association with the reference variables should be identical to within sampling error. The observed differences would be expected only if the two knowledge types are, in fact, measuring different constructs, which they seem to be. The most obvious difference is that local knowledge shows almost no association with the reference variables; Hispanic ethnicity being the only significant correlation. In contrast, general knowledge is significantly associated with all of the predictors.

**Table 9:** Correlations between reference variables and the watershed knowledge factors

Reference variable (high value)	General knowledge	Local knowledge
Sex (female)	–0.230***	0.062
Age	0.199***	0.091
Time in Locale	0.112+	0.019
Race (white)	0.192***	–0.011
Ethnicity (Hispanic)	–0.085	–0.163*
Education	0.206***	0.056
Income	0.160**	0.117+
Political Affiliation (conservative)	–0.055	–0.037

Note. *N* = 374. Correlations derived from the two-factor model with correlated errors

+ *p* < 0.10, *p < 0.05, **p < 0.01, ***p < 0.001

In summary, evidence of internal consistency was present for both indices in the form of positive and reasonably uniform loadings on their intended factor. And, crucially, the factor model accurately reproduced the observed correlations. Reliability was good for the general measure, but there remains room for improvement with respect to the index of local knowledge. The exploratory factor analysis produced some evidence of parallelism. Much stronger evidence emerged from the confirmatory analyses. The two-factor model with correlated errors showed excellent absolute fit to the data, superior fit to alternative models, and the ability to reproduce the observed correlations with the reference variables. All of these findings support the broader case for the construct validity of the measures.

### Study 3: Shorter and Longer Forms of the Watershed Knowledge Index?

#### RQ3: Index Length and Validity

As seen in Table 10, R values increase steadily as one moves across the table from left (two items) to the right (nine items). This is a quantitative illustration of the fact that as more elements of a fixed domain are added to the equation, the subset becomes an increasingly powerful predictor of the total.

**Table 10:** Regression models predicting general watershed knowledge from combinations of the nine items

Item	Regression Model (based on number of items)							
	2	3	4	5	6	7	8	9
**GD1**	0.389	0.269	0.181	0.144	0.099	0.074	0.026	0.000
**SC1**	0.451	0.349	0.288	0.230	0.214	0.185	0.202	0.167
**WF1**		0.454	0.376	0.337	0.269	0.212	0.185	0.182
**SC4**			0.368	0.320	0.281	0.274	0.274	0.214
**WF6**				0.296	0.293	0.245	0.212	0.204
**WF2**					0.259	0.236	0.217	0.198
**WF5**						0.225	0.190	0.178
**WF4**							0.172	0.184
**SC2**								0.181
*R*	0.732	0.840	0.899	0.937	0.963	0.981	0.990	1.000
^*Coefficient α*^	0.60	0.70	0.62	0.70	0.70	0.78	0.79	

Note. Entries in the body of the table are standardized regression coefficients (all *p* = < 0.001). The wording of the items is given in Table 3

The reliability data show a different pattern. The two-item short form presents a coefficient of 0.60, a value we view as the bare minimum. The remaining coefficients do not show a smooth increase because the inter-item correlation matrix—which partially determines α reliability—contains coefficients of varying magnitude. Inclusion of SC4 in the four-item index, for example, diminishes α because it is weakly associated with the other items. Consequently, the reliability data suggest natural cut-points for short forms. Three-items are a clear improvement over two-items (0.70 vs 0.60). But, after that there is no improvement until one reaches the seven-item index. Although the full nine-item version maximizes substantive validity *and* reliability, the three- and seven-item forms represent viable alternatives.

With only two items, the local knowledge index presents a different sort of problem. Shortening the measure would mean eliminating one or the other item. Because the close-ended item has a higher factor loading on the latent factor (0.73), it should be preferred if the researcher is forced to choose between them. But, the squared factor loading (0.53) can be interpreted as the reliability of that item (Brown 2015). This indicates that roughly half of the variance of the single-item measure is attributable to chance, a standard that is undesirably low. It is advisable to retain both items. Even that, however, gives a reliability coefficient of only 0.58. Hence, a better solution would be to include additional indices of local knowledge. From a conceptual standpoint, this is a simple matter: ask dichotomous questions regarding membership in other superordinate or subordinate watersheds. The Spearman-Brown Prophecy formula indicates that doubling the length of the index to four items would produce a reliability of 0.73. and tripling the number of items would yield 0.80 (Spearman 1904). As a practical matter, scoring the resulting data requires some degree of human evaluation, which imposes a different type of resource demand on researchers. This labor is seemingly unavoidable if the researcher seeks a more reliable measure.

### General Discussion

Collective identity is a prerequisite to collective action. We maintain that the development of collective identity is a stage process in which individuals acquire different sorts of knowledge, values, and experience. In the service of our theoretical and applied aims, the research considered three issues.

Because knowledge can be compromised in different ways, our first research question highlighted the presence of factual inaccuracies versus the absence of knowledge. In a sample of Pennsylvania residents, we observed low rates of inaccurate beliefs; fewer than 8% of the sample gave factually incorrect answers on the items we retained. The frequencies for *I don’t know* responses (i.e., lack of knowledge), were roughly double those for inaccurate beliefs. In other words, the absence of watershed knowledge was a much more common problem than the presence of inaccurate beliefs. This suggests that efforts at public education should prioritize the dissemination of new knowledge over changing existing, incorrect beliefs. This is surely good news for watershed management. Given the inherent durability of beliefs once they are formed (Nickerson 1998) and the relative ineffectiveness of interventions intended to change them (Walter et al. 2020), education is much easier than re-education.

Because neither theoretical nor applied research can proceed without valid measures, our second research question focused on the development of indices of watershed knowledge at three levels: 0 = No awareness, 1 = general knowledge, and 2 = general plus local knowledge. To address this issue the research team developed items, then consulted with subject matter experts and Pennsylvania residents. In combination, these data sources enabled informed scientific judgments of the substantive validity, internal consistency, and parallelism of the knowledge measures. The results established the two indices, *general* and *local* knowledge, of the Watershed Knowledge Index as distinct latent factors that can be assessed via unidimensional item sets.

With these tools, researchers can now reliably identify individuals as Stage 0, 1, or Stage 2 of collective watershed identity. Such data can contribute to resource allocation decisions concerning watershed policies, communication strategies, and programs intended to move a segment of the population to a higher stage of collective identity. For example, residents who in Stage 1—meaning that they understand the boundaries of a watershed—could be advanced to Stage 2 by informing them what watershed they reside within. Presumably, this local information would not be meaningful without the foundation of general knowledge.

Our third research question explored the relationship between index length and reliability of the two knowledge measures. We demonstrated that the general knowledge index could be shortened while acknowledging that acceptable levels of reliability may vary by field and by researcher. We recommend that the full item set be used whenever possible because it is the most valid option. However, we also appreciate that the nine-item index may be too resource-intensive for some projects. The three- and seven-item composites offer serviceable alternatives. The local knowledge measure was less reliable overall. Future research might supplement it with additional location items.

#### Implications for Theory Testing

The stage model of collective identity asserts that identity develops over time in a sequence of ordered categories (Mainzer et al. 2024). If this assertion is accurate, it suggests that efforts to create collective watershed identities—in the Chesapeake Bay Watershed or elsewhere—requires matching particular types of information to the members of each developmental stage. For example, individuals in Stage 0 need to acquire general knowledge about watersheds, whereas Stage 1 individuals would benefit from geographically specific information that locates them within various watersheds. This is a strong, but untested theoretical structure. Although the Watershed Knowledge Index enables a test of the structure at the early stages, a comprehensive empirical assessment will require purpose-built measures of Stages 3 and 4—work that is currently underway. However, there are several aspects of the current project that can be used to inform applied issues immediately. We comment on those next.

#### Watershed Knowledge Disparities

Health disparities are said to exist when social groups are differentially susceptible to certain health conditions or treatment is differentially available (Thorpe and Miller 2024). The set of sociodemographic indices used as reference variables reveal differences among social groups with respect to general and local knowledge. Hence, watershed knowledge disparities exist among Pennsylvania residents. Several factors were associated with relatively higher levels of general watershed knowledge: being male, being older, being white, being educated, and having higher income (Table 9). Being Hispanic was associated with relatively lower levels of local knowledge.

Although caution should be exercised before accepting any of the findings as facts, it is notable that they parallel health disparities: In this case, non-dominant or socially disadvantaged groups bear a disproportionate lack of knowledge. Perhaps, as was done in healthcare, special attention and effort should be expended toward reducing knowledge differences across social groups. If the stage model is correct, mitigating these disparities would enhance equity with regard to watershed knowledge and empower traditionally disadvantaged groups.

#### Implications for Watershed Managers

The state of Pennsylvania is divided into those regions that are inside of the Chesapeake Bay Watershed and those that are outside. Because our survey includes both, it enables comparisons that may be of use to watershed managers. Table 11 shows the number of survey respondents partitioned by location and stage of identity development. Column-wise comparisons for each row reveal two notable findings.

**Table 11:** Collective identity stages of participants in Pennsylvania

	CBW participants		Non-CBW participants	
	*(n* = *105)*		*(n* = *269)*	
Stage 0	20	19%	78	29%
Stage 1	20	19%	79	29%
Stage 2	65	62%	112	42%

First, we observed higher relative frequency of Stage 0 and Stage 1 respondents outside of the Chesapeake Bay Watershed versus inside (29% vs. 19% for both stages). If knowledge does move individuals toward collective identity and collective action, the need for education regarding foundational watershed concepts is more acute in Pennsylvania regions outside of the Chesapeake Bay Watershed. Second, there was a higher proportion of Stage 2 respondents inside (vs. outside) (z = 3.48, *p* = <0.001). Almost two-thirds of the inside sample possessed local watershed knowledge. This represents a notable messaging opportunity: A majority of residents are sufficiently knowledgeable that they should be responsive to messaging designed to move them toward consideration of environmental values (i.e., Stage 3) and the personal consequences of watershed degradation for themselves (i.e., Stage 4).

#### Limitations of the Stage Model

Because regional watersheds are physically large and socially heterogenous environments, it is likely that their residents hold a myriad of values, beliefs, needs, and positions. This diversity does not preclude development of a collective watershed identity though it may suggest limitations on movement across stages. Consider, first, that while the stage model identifies a path to collective identity, it does not promise that everyone will follow that path. Individuals might resist movement up the ladder of stages because pro-environmental values are incompatible with their political ideology, their livelihood, or their lived experience. The stage model is useful to the extent that it identifies groups of watershed residents who have certain features in common that make them receptive to messages tailored to their developmental phase. Second, it is important to bear in mind that everyone possesses many identities (e.g., farmer, friend, family member); adding a new one is not necessarily difficult. People presumably move toward a collective identity when they understand that they share a common history and common future with others. How to make a watershed identity salient relative to all the other competing identities is a challenge for future research.

We argue that access to clean water is a common need for all. Yet we acknowledge that this need is framed by a person’s role in the watershed as, for example, a farmer, a parent, a conservationist, or many other overlapping characteristics. This reality highlights the need for clear audience identification in any future application of the model. Our data suggest existing disparities in watershed knowledge in Pennsylvania along the lines of sociodemographic characteristics—namely the gap between older, educated, white males, and Hispanic people. But more work is necessary to accurately define audience segments and, critically, that work must be locally sensitive to a specific watershed.

We wish that all people would care about water quality as a basis for human health and well-being. However, the ways in which they may demonstrate that care through actions are complex and nuanced. Our proposed stage model simplifies this process somewhat. Like voting, people are expected to make simple decisions—one candidate or the other—that behold an intricate set of information. The presence of political parties and their associated platforms help to simplify this decision in most cases to a binary decision (Mainzer 2025). Our stage model follows a similar approach in that a few criteria, general knowledge and local knowledge, are needed to become aware of group membership. This has the benefit of parsimony for researchers and managers at the expense of specificity.

## Conclusion

The findings described herein should be seen as a useful beginning for researchers, managers, and activists interested in developing communication strategies for pro-environmental behavior changes in Pennsylvania. However, we hope that the stage model and its associated knowledge measures can be applied more broadly, across geographic and civic boundaries. We view them as tools that are essential to understanding the role of collective identity in watershed management and, therefore, the basis for collective action.

## Data Availability

Data may be made available upon request, but is not currently available for public use.

## Footnotes

1 David and Bar-Tal (2009) draw clear and helpful lines between these two phrases. They argue that social identities exist as beliefs (in the heads of individuals), whereas collective identities exist in the communication behavior that occurs between social actors (i.e., individuals, institutions, or media). Because we believe that there is considerable empirical overlap between them, we intentionally gloss those distinctions in this paper.

2 One reviewer wondered why no social scientists were included as subject matter experts. At the time of study design, our attention was focused on expertise in ecology. Without much reflection, it was assumed that the social science expertise of the research team members was adequate. In retrospect, the formative phase of research might have benefitted from the objectivity of third-party social scientists.

3 To guard against question order effects, items were randomized within blocks, where the blocks were defined in terms of item content (i.e., general definition followed by spatial concepts followed by watershed functions). Block ordering was determined by logic: it is possible to correctly answer the general definition question and still fail the later questions. And, in the judgment of the research team, function questions (why?) represent more sophisticated knowledge than spatial questions (what?).
